# Editorial: Individual versus Dyadic Processes: Health and Relationship Outcomes

**DOI:** 10.3389/fpsyg.2021.714548

**Published:** 2021-07-21

**Authors:** Maria Nicoleta Turliuc, Anne Milek, Tea Trillingsgaard

**Affiliations:** ^1^Department of Psychology, Alexandru Ioan Cuza University, Iaşi, Romania; ^2^Department of Psychology, University of Münster, Münster, Germany; ^3^Department of Psychology, Aarhus University, Aarhus, Denmark

**Keywords:** individual processes, dyadic processes, health outcomes, relationship outcomes, interdependency

## Introduction

Building strong relationships is a fundamental human need, and finding an intimate partner is evolutionary important for survival and procreation (Buss and Schmitt, 1993). Once established, intimate relationships entail interpersonal support processes that are fundamental to growth, development and coping with life's adversities (Feeney and Collins, 2015). Intimate partners have a strong mutual influence over on each other's health and stress experiences (Randall and Bodenmann, 2017; Sbarra and Coan, 2018). The number and quality of intimate relationships are associated with many health outcomes including immunological and endocrine responses (Hostinar et al., 2014), cardiovascular disease and cancer (Farrell and Stanton, 2019) as well as length of life (Holt-Lunstad et al., 2017). At the same time, discord in intimate relationships is involved in the onset, severity, and progression of a wide range of diseases, as well as in the severity, progression, treatment, and recovery from mental health disorders (Dunkel Schetter, 2017). Research consistently indicate that individual processes and conditions may also affect health. Most often studied, the big five personality traits interact to predict sexual health (Allen and Walter, 2018) or mental treatment outcomes (Bucher et al., 2019). Considering illness as an individual cognition, Singer and his colleagues (Singer et al., 2010) found that one-third of the cancer patients in acute care hospitals is suffering from mental health disorders, depression being the most common psychiatric condition (Singer et al., 2010). Moreover, individual processes and conditions may also affect relationship outcomes. For example, self-reported and partner-perceived reported personality traits (Weidmann et al., 2016), attachment insecurity (Candel and Turliuc, 2019), emotional regulation (Bloch et al., 2014) or emotional intelligence (Malouff et al., 2014) were found to play important roles in predicting relationship satisfaction. Finally, various dyadic processes are important predictors of relationships outcomes. Yoo et al. (2014) found that sexual satisfaction significantly predicted emotional intimacy, and that both variables mediated the association between spouse's communication and their own relationship satisfaction, for both husbands and wives. Relationship stress is a mediator between external stress and marital communication or marital quality (Ledermann et al., 2010). Further, marital communication mediates the association of relationship stress with marital quality. Also, dyadic coping strongly predicts relationship satisfaction regardless of gender, its aggregated positive forms being a stronger predictor of relationship satisfaction than the aggregated negative ones (Falconier et al., 2015). Systematically analysing both individual and dyadic processes, Joel and her colleagues (Joel et al., 2020) used machine learning techniques to predict relationship quality across 43 dyadic longitudinal datasets of 11,196 romantic couples (Joel et al., 2020). Their findings indicate that the top individual-difference predictors of relationship quality were life satisfaction, negative affect, depression, attachment avoidance, and attachment anxiety, and the top relationship-specific predictors of relationship quality were perceived-partner commitment, appreciation, sexual satisfaction, perceived-partner satisfaction, and conflict (Joel et al., 2020).

## Key Premisses of the Research Topic

Together with individual differences (e.g., personality traits, attachment dimensions, positive and negative emotions, illness etc.), dyadic processes (e.g., perceived and received support, self-disclosure, dyadic coping, dyadic emotion regulation, conflict, forgiveness, etc.) influence both the individual's health (physical and the psychological well-being), as well as the quality of his/her intimate relationship (e.g., intimacy, commitment, love, and relationship satisfaction). Consistent research findings indicating that close relationships (with the romantic or marital partner) are one of the longer-term, more salient, and mutually influential relations. On one hand, there is evidence indicating that individual differences shape people's health, psychological well-being, and their close relationship. On the other hand, research findings also indicate that intimate relationships may affect health through biological, behavioural, and psychosocial pathway, shape health and relationship outcomes throughout the life course and have a cumulative impact over time. Moreover, what happens inside couples' life is important because intimate interaction and co-regulation impact relationship' quality and well-being.

## This Special Research Topic

Based on these findings and premisses, understanding the interrelatedness between close relationships, health and well-being becomes even more crucial. We need research to disentangle the specific dyadic behaviours or interaction patterns that underlie this interrelatedness. We also need to focus on the large heterogeneity in how relationships fare in terms of health and functioning over time and to understand the individual differences in circumstances, traits or states that may explain this variability. Finally, we are in constant need for improved methods to design dyadic studies, sample data of better quality and improve tools to model the complexities of dyadic data. The current Research Topic features contributions from numerous esteemed researchers who offer a variety of high-quality, informative publications on these key issues in the field of close relationships. We present original empirical reports, literature reviews, and demonstration of novel developments within research methodology. It is our hope that the articles included in this collection will inform readers about the latest developments in the field, inspire the development of theory and methods to understand relationship dynamics and stimulate discussions about effective interventions to support strong relationships in practise.

In this topic, you will find that several notable themes emerged throughout the 17 contributions. One first central theme is the role of support provision and receipt and its consequences for health and relationship outcomes. Scholz et al. reported from three rigorous diary studies on the way positive and negative social control from a partner influences own affect and health related behaviour. Song et al. included both quantitative and qualitative data describing the crucial role of lay caregivers in the survival of patients after allogenic hematopoietic stem cell transplant, a patient group exposed to an extensive and demanding self-care regime. To investigate the thought-provoking idea that support provision is beneficial for the support provider, Berli et al. conducted a study on couples dealing with overweight and inactivity and examine the association between providing support, physical activity, affect and relationship satisfaction. The idea that shared pursuit of goals has positive implications for both the individuals and the relationship was also explored by Ungar et al. In their study, they investigate whether joint goals in older adults (and an accurate perception of what is joint or not) was associated with goal progress, relationship satisfaction and nine different biomarkers summed up to report the allostatic load. Stefǎnut et al. systematically assessed the results of previous research on the relationship between dyadic coping and emotional well-being as well as the relationship between dyadic coping and the relationship quality in cancer patient and their partners. Support provision, support receipt and we-perspective on burdens and joys of life are all key tenets of theoretical models within relationship science and the current articles in this topic offer unique perspectives on these dynamics.

A second theme throughout this topic is the way in which physical or mental health, as individual conditions, shape the interpersonal process. Rapelli et al. presented a study on married couples faced with cardiac illness in which they examined the dyadic coping strategies as a potential moderator of the link between perceived distress and partner support. Bertschi et al. systematically identified, selected, and critically discussed previous research to describe the key dyadic challenges and dyadic coping strategies when one partner has a chronically disabling physical or sensory impairment. Nalbant et al. investigated the reasons of separation in partners or ex-partners of cancer patients, the factors influencing separation, and the positive or negative perception of the impact of cancer on the relationship. Overall, this group of pieces contribute with important insights into the dyadic challenges of coping with illness, disability or distress.

A third theme in this topic is on the heterogeneity of health and relationship thriving caused by the history, circumstances, and experiences of the individual, including both state and trait characteristics. Zuo et al. used three datasets with heterosexual couples to investigate the concurrent and longitudinal association between trait self-control and romantic relationship satisfaction, also when controlling for commitment. Candel and Turliuc reported from an online daily diary study on how partners' sense of relational entitlement affects day-to-day couple satisfaction levels in interaction with variables of the interpersonal process model of intimacy. Celsi et al. examined childhood-related predictors and mediators of cyber dating abuse among young non-cohabiting partners. With a 5-wave longitudinal dataset tracking newlywed couples along the years, Kuile et al. investigated how pre-pregnancy happiness in the relationship functions as a predictor of post-natal changes in relationship commitment for fathers and mothers, in comparison with childless couples. Going from dating, newlywed, and new parenthood couples to the other end of the relationship cycle, Sander et al. examined data from recently divorced men and women. In their study, they seek to understand both overall levels of mental and physical health in the divorce population, as well as the individual differences in response to divorce as predicted by conflict levels, objective circumstances of the divorce and relationship history. Horn et al. conducted an online diary study to understand the interplay of intrapersonal emotion regulation (rumination) with interpersonal regulation processes (disclosure quality) in the context of the adjustment to retirement in late adulthood. As highlighted by studies on this theme, both trait and state of the individual is an important context for understanding relationship outcome and health.

A final contributing theme of this topic arises from the articles that pursue methodological issues for standardised lab paradigms. Liekmeier et al. applied a novel method for modelling microlevel observed changes in affective behaviour during a discussion task, using data obtained from two in-therapy parent couples with different slopes of change during a discussion task, a potentially important marker of response to therapy. Pauw et al. investigated the often neglected but potentially influential spill over of lingering affect from one experimental task to the next when partners are instructed to take turns in providing support to one another. To take a step forward in the dynamic modelling of physiological data from the lab, Li et al. explored patterns of physiological linkage in cardiovascular data from male same sex partners interacting around trivial as well as sensitive discussions of health and appearance in the lab. These studies contribute to the current developments of the lab paradigm and to the improvement of this important setting for collecting dyadic data.

This collection of research put forward the central idea of dyadic interdependency, support, and co-regulation, yet all contributions differ markedly in the choice of time unit resolution: from one time-frame measurement to multiple waves of data collection, from weekly daily diary sampling to moment-to-moment fluctuations within minutes in the lab. Surely, the coregulation within close and caring relationship occur at all these time levels, and at all developmental stages. The articles in this collection span from college students engaged in (cyber)dating, young adults going through the newlywed and early parenthood years, couples coping with poor health conditions (obesity and physical inactivity, cancer, cardiac illness), male same sex couples in the lab, parental couples in therapy, divorcing couple with and without new relationships, couples transitioning to retirement, and couples in old age. The dyadic processes in close and caring relationship are linked to health and well-being at all stages of the life span.

## Conclusion and Special Thanks

In conclusion, the studies presented in this Research Topic provide a comprehensive and cutting-edge view of the ways in which individual and dyadic processes act and interact in shaping health and relationship outcome. The articles indicate some of the most promising ways of approaching this topic, include the combination of individual and dyadic perspectives and the modelling of data interdependency using state-of-the-art research methods.

We are grateful to all authors of this special issue for sharing their significant scientific contributions and to the many peer-reviewers who provided great knowledge and feedback on them. This Research Topic calls upon our community of researchers working with close relationships to adapt study designs that are even better in capturing and analysing the interdependent processes between dyad members in the lab, in diary studies, and in surveys. It calls upon both researchers and clinicians to attend—in research and practice–to the contextual circumstances, traits and states that shape the large variability in how relationship changes. Finally, this topic calls on us to continue to foster relationships with feelings of intimacy, we-ness, responsive support, and acceptance. This are of particular importance across those many life stages when relationships are at stake, under change, and the key source of support.

## Author Contributions

All authors listed have made a substantial, direct and intellectual contribution to the work, and approved it for publication.

## Conflict of Interest

The authors declare that the research was conducted in the absence of any commercial or financial relationships that could be construed as a potential conflict of interest.
